# Different Lower-Limb Setup Positions Do Not Consistently Change Backstroke Start Time to 10 m

**DOI:** 10.3390/sports8040043

**Published:** 2020-03-31

**Authors:** Gordon E. Barkwell, James P. Dickey

**Affiliations:** School of Kinesiology, University of Western Ontario, London, ON N6A 3K7, Canada; gbarkwel@uwo.ca

**Keywords:** biomechanics, swimming, backstroke start, position, electromyography, muscle activation

## Abstract

Backstroke starts involve the athlete starting from a flexed position with their feet against the pool wall and then extending their ankles, knees, hips and back to push off; however, swimmers can start in different positions. The purpose of this study was to evaluate the performance impact of different knee extension angles in the setup position for a backstroke start. Ten backstroke swimmers completed maximum-effort starts in each of two setup positions: one with the knees maximally flexed, and one with the knees less flexed. The start handles and touchpad were instrumented with multi-axial force sensors. Activity of major hip and knee extensors was measured using surface electromyography. Body position in the sagittal plane was recorded using high-speed cameras. There was no overall difference in time to 10 m between the two conditions (*p* = 0.36, d_z_ = 0.12), but some participants showed differences as large as 0.12 s in time to 10 m between start conditions. We observed that starts performed from a setup position with less knee flexion had an average 0.07 m greater head entry distance (*p* = 0.07, d_z_ = 0.53), while starts from a setup position with maximal knee flexion had an average 0.2 m/s greater takeoff velocity (*p* = 0.02, d_z_ = 0.78). Both head entry distance and takeoff velocity are related to start performance, suggesting each position may optimize different aspects of the backstroke start. Coaches should assess athletes individually to determine which position is optimal.

## 1. Introduction

In 2014, the backstroke ledge was approved by FINA (Fédération internationale de natation, or International Swimming Federation) for use in competition. The backstroke ledge alters the angle as well as the coefficient of friction between the feet and the starting surface [1], fundamentally changing backstroke start performances [2,3,4]. Accordingly, findings from previous studies where the backstroke ledge was not used may no longer be relevant. Since use of the backstroke ledge appears to offer a performance advantage [2,3,4,5], it has been almost universally adopted as the preferred starting surface. Two studies have analyzed the impact of different handgrip positions while using the backstroke ledge; one determined that it does not affect start time [4], and the other did not discuss start time [6]. No other analysis of setup technique has been performed on starts where the backstroke ledge is used.

Performance implications of initial joint angle selection have not previously been analyzed in backstroke starts, but differences in lower-limb setup position have been noted between backstroke swimmers [3]. Squat jumps, which are somewhat similar to backstroke starts, may provide some insight regarding the impact of different setup positions. Computer modeling of squat jumps reveals peak jump height is achieved when a deeper initial squat position is selected [7,8]. Interestingly, human participants do not show a difference in maximum jump height, but a longer contact time from a deeper squat. This was perhaps because participants were less accustomed to the deep squat position [9] and thus were unable to achieve the optimal muscle activation sequence for that starting position [8]. Additionally, a proximal to distal pattern of joint extension is generally assumed to aid in achieving a high takeoff velocity. This order of joint extension has been found related to greater performance in computer simulations of vertical jumps [7,8,10], jumps performed by participants [8,9] as well as in backstroke starts [2,11]. However, it appears that this order of joint extension is not always achieved in the backstroke start, since some studies have observed no differences in joint sequencing between starts with and without the backstroke ledge while observing improvements in start time [3,4]. Therefore, it is important to explore start techniques that may result in a proximal-to-distal strategy.

Muscle slack can also affect performance in explosive movements and should be considered in the backstroke start. Muscle slack is represented by the delay between the onset of muscle contraction and recoil of the series elastic elements, which increases response time [12]. Pretension through co-contraction of agonist-antagonist pairs is suggested as a method for reducing muscle slack [12]. In the backstroke start, this type of pretension may be achieved by starting with the lower-limbs in a more extended position. In this start position, swimmers hold themselves further above the water and further out from the pool wall by contracting their lower-limb extensors. Gravity pulls the swimmer down while contraction of the extensors pushes them up, effectively removing extensor muscle slack in the same fashion as co-contraction.

Electromyography (EMG) is a useful supplement to kinematic data because it provides a detailed picture of the muscle forces which are resulting in the joint movement. It is not often implemented in swimming due to the challenges associated with waterproofing the instrumentation [13,14,15]. Nevertheless, EMG has been used to determine muscle activation patterns in backstroke starts [16], as well as to compare differences between different start conditions [17,18]. It is important to understand both timing and intensity of muscle activity; however, a recent review indicates that most studies only report intensity and that analysis of timing is a significant gap in the literature [13].

In ventral swimming starts, different setup positions [19,20], forward or rearward initial leaning [21], and entry angles [22] have been evaluated. In contrast, the backstroke start has received relatively little attention. In existing backstroke start literature, different methods and analysis techniques make direct comparison between studies difficult [13]. Furthermore, most studies which discuss different start configurations [18,23,24,25] were performed prior to recent equipment changes, and thus their findings may no longer be relevant. The few studies which discuss different configurations while using the backstroke ledge only manipulate hand position [4,6]. Accordingly, the purpose of this study was to compare two different backstroke start setup positions and determine if one results in better performance than the other. Our primary outcome measure was time to 10 m, which has been used to evaluate start performance in previous backstroke start studies [3,17,23]. We did not measure multiple distances since previous work has identified that time to 5 m, 10 m and 15 m all had statistically significant differences of the same polarity between two start conditions [23]. Our secondary outcome measures were joint kinematics, head entry distance, and muscle activation. It was hypothesized that starts performed with the lower-limbs in a more extended position would result in improved performance due to increased muscle activation prior to the start, a more proximal-to-distal joint extension strategy and a lower wall contact time.

## 2. Materials and Methods

This study was approved by the Western University Health Sciences Research Ethics Board and all participants provided written informed consent. Participants were backstroke swimmers from the same varsity swim team and were eligible if they scored 600 or more FINA points in a backstroke event. A power analysis was performed using commercially available software (G*Power version 3.1.9.3, Department of Psychology, Heinrich Heine Universität, Duesseldorf, Germany) [26] based on our primary outcome measure and assuming that the difference between start conditions would have the same effect size as the difference between starts with and without the OBL2 [3]. This analysis determined that ten participants were required for 80% power to detect an effect size of 0.89 using a one-tailed repeated measures *t*-test with a probability of 0.05. All swimmers were regularly training at the time of data collection. Data collection coincided with the start of the training taper prior to major competitions. Participants were free of injury at the time of data collection.

**Figure 1:**
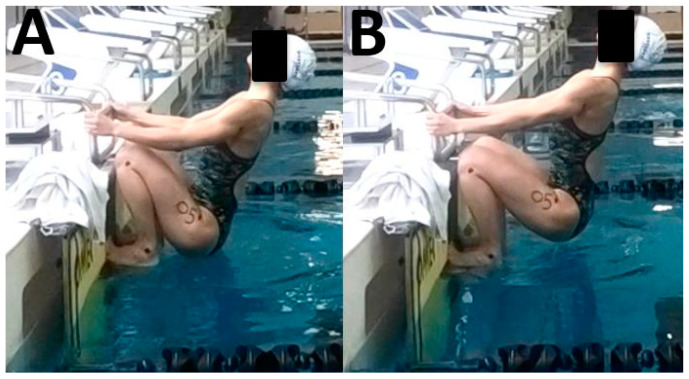
Body positions in the flexed (**A**) and extended (**B**) setup positions as illustrated in this representative participant.

Testing was performed in an indoor 50 m pool. All participants completed the same warm-up, which included 900 m of swimming and drills as well as two practice starts. This procedure was consistent with this swim team’s typical pre-meet warm-up for a sprint backstroke race. Swimmers then performed two more maximum-effort starts from which data were collected for this study. All starts were compliant with FINA guidelines, meaning swimmers were stationary prior to the start signal and surfaced before the center of their head reached the 15 m mark [27]. Participants were instructed to complete race-pace starts to a minimum of 15 m, including maximum effort underwater kicking. In one start, participants had maximum knee flexion in the “take your mark” position: participants were told to bring their hips close to their ankles (Figure 1A). In the other start, participants’ knees were more extended in the “take your mark” position: participants were told to push their hips out from the wall (Figure 1B). Participants practiced both setup positions prior to their practice starts. The order in which participants performed the two start types was determined based on their study-specific code such that half of the participants started with the flexed setup position and half started with the extended setup position. Two minutes of rest were given between starts, which has been found to be an adequate recovery period for backstroke starts [3,4,17,18,23,25].

**Figure 2:**
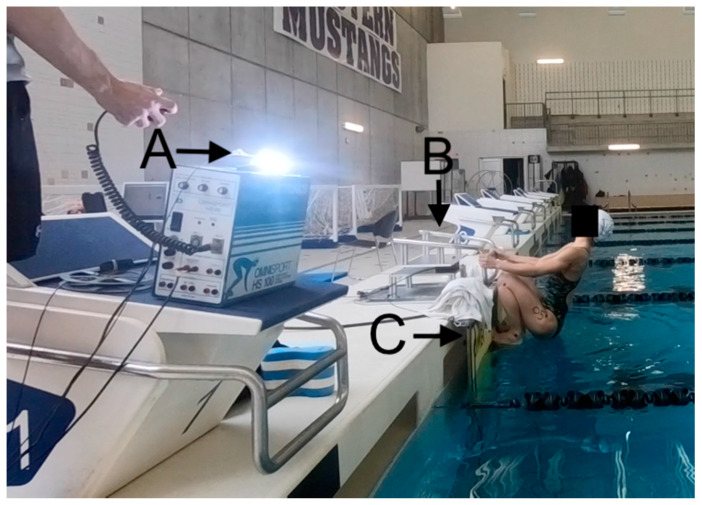
Experimental setup for this study at the instant of the start signal with the swimmer in the flexed start position. (**A**): Starter strobe and fiber optic cable carrying the light to the underwater camera. (**B**): Omega OSB11 start handles mounted to load cell. (**C**) Backstroke ledge (Omega OBL2) and touchpad (OCP5) mounted to waterproof force plate.

The pool bulkhead was instrumented with a tri-axial waterproof force plate (OR6-WP-2000, AMTI, Waterdown, MA, USA), which was faced with an ultra-high molecular weight polyethylene sheet (EZ-Glide 350, Eclipse Sports, Cambridge, ON, Canada). To simulate race conditions, a FINA standard touchpad (Omega OCP5, Swiss Timing, Corgemont, Switzerland) and backstroke ledge (Omega OBL2, Swiss Timing, Corgemont, Switzerland) were attached directly to the polyethylene sheet so that all forces registered on the force plate. A set of backstroke handles from a FINA standard start block (Omega OSB11, Swiss Timing, Corgemont, Switzerland) were attached to a load cell (Omega 160, ATI, Apex, NC, USA) and mounted to the top of the pool bulkhead with the handles aligned with the surface of the touchpad, which is consistent with FINA guidelines [28]. The equipment setup is pictured in Figure 2.

Two wireless surface electromyogram (sEMG) sensors (Trigno, Delsys, Boston, MA, USA) were fixed to the participants’ skin over the right vastus lateralis and gluteus maximus muscles using double sided adhesive from the manufacturer (SC-F03, Delsys, Boston, MA, USA). These muscles were selected to represent the action of the knee and hip extensors, respectively. Sensor locations and orientations were determined based on recommendations from the SENIAM project [29]. Sensors were covered with an adhesive film (Tegaderm^TM^, 3M^TM^, St. Paul, MN, USA) to prevent water intrusion. One end of a 15 m length of RG-174 coaxial cable (GoPro Underwater WiFi-View, Eye of Mine action cameras, Long Beach, CA, USA) was taped beside the gluteus maximus sensor to carry the wireless signal above water when that sensor was submerged.

All starts were recorded from the side using two high-speed digital video cameras (Hero 6 Black, Go Pro, San Mateo, CA, USA) recording in 1080p at 120 frames per second with a 1/480 s shutter speed. Both cameras captured the left side of each participant. One camera was located above water, 1 m from the end wall and captured the entire above-water portion of the start. The second camera was located underwater, 10 m from the end wall and was used to measure the swimmers’ time to 10 m. Cameras were started synchronously using a WiFi remote (Smart Remote, Go Pro, San Mateo, CA, USA), and the signal from this remote was carried to the underwater camera using a 15 m length of RG-174 coaxial cable (GoPro Underwater WiFi-View, Eye of Mine action cameras, Long Beach, CA, USA). A competition starter (Daktronics, Brookings, SD, USA) was used, which provided an audible start signal for the swimmers and a flash for timing [28]. Synchronization of the video files was verified by visualizing the start strobe light at the start of the video recordings, which was transmitted to the underwater camera via a length of fiber optic cable (810004, Industrial Fiber Optics Inc, Tempe, AZ, USA). To ensure consistent digitizing of joint coordinates, anatomical landmarks were marked with 1 cm diameter circles using eye black (Easton Baseball/Softball Inc., Van Nuys, CA, USA) at the left ankle (lateral malleolus), knee (lateral epicondyle of the femur), hip (greater trochanter) and shoulder (greater tubercle). Joint locations in the video files were manually digitized using Kinovea software (Version 0.8.25, (Kinovea open source project, www.kinovea.org). All files were digitized by the same individual. The hip extension angle was calculated from the position of the knee, hip and shoulder markers while the knee extension angle was calculated from ankle, knee and hip markers; this method of calculating hip and knee angles is consistent with previous work [2,11]. Hip and knee angles in the setup position were recorded at the instant of the starter flash. To reduce noise from manual digitizing error, joint angle waveforms were zero-lag filtered with a 2nd order low-pass Butterworth filter. Residual analysis indicated that a 4 Hz cut-off frequency was optimal [30]. Onset of hip and knee extension were calculated from the filtered waveforms. Average hip and knee angular velocities (degrees/s) were calculated by dividing the change in joint angle (degrees) by the time elapsed between the starter flash and last wall contact (s). Onsets were automatically calculated using a published algorithm [31] and visually inspected for accuracy. Head entry distance was calculated as the distance between the front surface of the touchpad and the center of the head as it entered the water. Time to 10 m was calculated as the time elapsed between the starter flash and when the center of the head reached 10 m, indicated by a weight line located at the 10 m mark on the opposite wall from the underwater camera, and clearly visible in the video image. The position of the center of the head was selected, rather than other measures such as whole-body centre of mass [32], as it is used in competition to determine start infractions [27].

To normalize muscle activity, each participant performed maximum voluntary isometric contractions (MVICs). Three repetitions of four second duration MVICs were collected for each muscle [33]. A two-minute break was provided between MVICs, and between the last MVIC and the first backstroke start. For vastus lateralis, participants were seated with 90 degrees of hip and knee flexion and attempted to extend their right knee while the ankle was restrained by a cuff and chain [33]. For gluteus maximus, participants were prone with 90 degrees of hip and knee flexion and attempted to extend their right hip while the distal portion of the thigh was restrained by a cuff and chain [33]. Participants were given verbal encouragement during all MVICs to promote maximum effort.

Voltage signals from the sEMG, load cell, force plate and starter were sampled synchronously at 1000 Hz using a 16-bit analog-to-digital conversion board (USB-6225, National Instruments, Austin, TX, USA) and recorded using a custom LabVIEW program (Version 2010, National Instruments, Austin, TX, USA). Force plate and load cell signals were converted to forces and moments using calibration matrices from the respective manufacturers. Calibration was verified using a digital force gauge (M5-500, Mark-10, Copiague, NY, USA). The time integrals of the forces were calculated to obtain horizontal and vertical impulses of the hands and feet. Forces were integrated between the reaction time (when forces first changed after the start signal) and last contact (when the forces reached zero). Impulse-momentum theorem was then applied to calculate predicted horizontal and vertical takeoff velocities. Additionally, the magnitude and trajectory of the net takeoff velocity was calculated at the end of the block phase from the predicted horizontal and vertical velocities. Peak rate of force development through the feet was calculated using the derivative of the net force-time curve from the force plate. Hand and foot contact times were also calculated using the force data as the time elapsed between the start signal and when the forces on the handles and wall reached zero, respectively.

sEMG voltages were processed to linear envelope EMG using a custom LabVIEW program (Version 2013, National Instruments, Austin, TX, USA). Voltages were full-wave rectified, then filtered using a second order 0.1-3 Hz band pass Butterworth filter. The time delay created by the 2nd order Butterworth filter is comparable to the electromechanical delay of the muscle so the linear envelope EMG represents muscle force [34]. The resulting waveforms were then expressed as %MVIC. From these data, the average muscle activation during setup (averaged over the 0.5 s immediately prior to the start signal), peak activation during the block phase, time to peak activation during the start, time of muscle activity onset over baseline, and predicted average rate of force development during the start were calculated for each muscle. Onset of muscle force was calculated automatically using a published algorithm [31] and each trial was visually confirmed to ensure accuracy.

Data were analyzed using paired, one-tailed *t*-tests with an alpha value of 0.05. The effect size for repeated measures experiments (Cohen’s d_z_) was also calculated for each comparison [35]. For effect size, standard thresholds of 0.5 and 0.8 were used to define medium and large effect sizes, respectively [35]. Pearson’s correlation coefficient was calculated to determine the strength of the relationship between head entry distance and start time. We defined a correlation coefficient of 0.3–0.5 as “fair”, 0.6–0.8 as “moderately strong” and greater than 0.8 as “very strong” [36]. The reliability of manually digitizing the anatomical landmarks was assessed for each of the four landmark positions using the coefficient of multiple determination (R^2^_a_) for one trial that was digitized three times [37].

## 3. Results

Ten swimmers (seven females and three males, 19.2 ± 1.4 years old) participated in this study. The average FINA point score across the participant pool was 695.5 ± 37.8 points based on each participant’s highest FINA point score in a backstroke event.

The coefficient of multiple determination confirmed there was little variability in the manual digitizing of the joint coordinates. The ankle, knee, hip and shoulder joints had R^2^_a_ values of 0.86, 0.995, 0.998 and 0.994, respectively. For all participants, the initial knee angle was significantly greater (an average of 18.7° greater) in the extended start position (*p* < 0.01, d_z_ = 4.79). The position of the hip relative to the surface of the water was an average of 0.04 m higher in the extended start position (*p* < 0.01, d_z_ = 1.09). Onset of knee extension occurred an average of 0.02 s later in the extended start (*p* = 0.02, d_z_ = 0.75). Head entry distance was, on average, 0.07 m greater in the extended start (*p* = 0.07, d_z_ = 0.53). Additionally, there was a fair negative correlation (r = −0.51) between head entry distance and time to 10 m which was statistically significant (*p* = 0.02). There was also a fair negative correlation between time to 10 m and the horizontal (r = −0.45) and net (r = −0.46) takeoff velocity. These correlations were also statistically significant (*p* = 0.043 and 0.037, respectively). The remaining kinematic variables were not significantly different between test conditions and had small effect sizes (Table 1). Kinematic data for the individual participants are presented in Appendix A, Table A1.

The vertical impulse applied through the hands was an average of 14.3 Ns higher (*p* = 0.02, d_z_ = 0.81) during the flexed start. Horizontal takeoff velocity and net takeoff velocity were also higher in the flexed starts by an average 0.18 (*p* = 0.03, d_z_ = 0.66) and 0.20 m/s (*p* = 0.02, d_z_ = 0.78), respectively. The remaining kinetic variables were not significantly different between test conditions (Table 2). Kinetic data for the individual participants are presented in Appendix B, Table A2.

On average, the vastus lateralis activity prior to the start signal was more than two times higher in the extended start than the flexed start (average increase of 12.5% maximum voluntary contraction (MVC); *p* < 0.01, d_z_ = 1.05). The remaining EMG variables were not significantly different between test conditions (Table 3). EMG data for the individual participants are presented in Appendix C, Table A3.

## 4. Discussion

This study investigated whether a specific lower-limb setup position was related to better start performance. The results revealed that there was no statistically significant difference in time to 10 m between start conditions, failing to support the hypothesis that setup with the lower-limbs in a more extended position would result in a shorter time to 10 m. This indicates that there is no performance advantage in selecting one setup technique over the other. However, differences in kinetics, kinematics and muscle activity between the two start conditions present relevant information regarding backstroke start performance.

Horizontal takeoff velocity, total takeoff velocity and vertical impulse through the hands were significantly higher in the flexed start. These data suggest that the flexed start position results in better performance during the block phase (between the start signal and last contact with the wall). Since contact time was not significantly different between conditions, this difference in impulse is likely due to a change in the magnitude of the applied forces. These findings are comparable to previous research which compared above and below water foot positioning; starts with the feet placed above the water had a greater horizontal impulse and takeoff velocity but no significant difference in time to 10 m [24]. These seemingly contradicting data highlight the importance of considering all phases of the start, and not simply the block phase.

As expected, participants had a significantly greater initial knee extension angle in the extended start condition. This confirms that participants were consistently adopting different knee angles in the two setup positions. This change in knee angle meant that the swimmers held their hips higher above the surface of the water in the extended start position. The onset of knee extension was significantly later in starts performed from a more extended position; however, there was no statistically significant difference in time of hip extension onset, hip angular velocity or knee angular velocity between conditions. This indicates a more proximal-to-distal order of joint extension when employing the extended start position, and further indicates that this setup position resulted in better performance during the block phase.

Vastus lateralis activity was an average 12.5% higher during setup in the extended start. This is because a knee extensor moment is needed to hold the body further above the water. The greater vastus lateralis activity observed in the extended setup position suggests there was also a greater muscle force, and thus less knee extensor slack for these starts. However, this did not translate to a lower wall contact time as hypothesized. There was also no statistically significant difference in peak vastus lateralis activity between conditions, indicating that the higher activity prior to the start did not promote greater force production later in the block phase.

Some studies analyzing dive starts have used kinetic block performance to predict time to 2 m [38,39]. However, this calculation is not appropriate in the backstroke start since, in most cases, there is drag from the feet and lower legs passing through the water during the flight phase. Head entry distance has been implemented in previous studies to describe block phase performance, and the magnitudes observed in this study are consistent with those observed by other researchers [5,23]. Participants had a greater head entry distance (medium effect size) when starting from a more extended position. This suggests that the extended start position resulted in better performance during the flight phase. This may be explained by the higher hip position during setup in the extended position, suggesting that the center of mass (COM) was higher as well. A higher COM position may result in a flight phase with less drag [23]. The more proximal-to-distal order of lower-limb joint extension observed in the extended start may also have contributed to this difference.

Despite the flexed start resulting in better block phase performance, and the extended start resulting in better flight phase performance, we observed no significant change in time to 10 m between conditions. Analysis of relay takeover techniques reveals similar findings: a single step technique results in a greater entry distance and a double step technique results in a greater takeoff velocity, but there is no significant difference in time to 10 m between the techniques [40]. Start time (such as time to 10 m) is not easily captured by coaches; however, it is a direct measurement of start performance. Correlations can help identify variables that are closely related to time to 10 m and may be more easily captured by coaches. Our past studies that examined backstroke start equipment changes [3] and warm-up modifications [17] found strong correlations between head entry distance and time to 10 m. Studies completed by other researchers have also shown lower start times which are associated with increased entry distances [5,23]. Although the correlation was only fair in this study, it still supports the relationship between head entry distance and time to 10 m. Accordingly, it is clear that the relationship between head entry distance and start time holds across a wide range of situations.

This study had several limitations, and results should be interpreted accordingly. The participants performed maximum-effort swim starts to 15 m in this experiment, which was a routine component of their training and racing, but may be different than their performance in races. We also only captured time to 10 m. However, this was appropriate as it isolates the start from the free-swimming portion of the race, and previous research has identified that time to 5 m, 10 m and 15 m all had statistically significant differences of the same polarity between two start conditions [23]. The fact that ankle joint kinematics were not captured is another limitation. This was because the ankle was partially submerged during the start for many participants, and thus we were not able to accurately quantify ankle angles. We estimated the rate of force development for the gluteus maximus and vastus lateralis muscles based on EMG data, which is appropriate since linear envelope EMG closely resembles muscle force [34,41]. While this approach accounts for the muscle activation-dependent active components muscle force, it does not account for force produced by passive properties of the muscles and tendons [42]. Since force plate data only describe net forces, we estimated muscle force from EMG to provide a muscle-based metric of rate of force development despite the limitations.

Another limitation was that males and females were grouped in the analysis. We minimized the impact of this limitation by implementing a repeated measures design. Accordingly, differences between males and females, such as males producing a higher muscular power per unit bodyweight [43] and having greater drag in backstroke starts [44] did not affect our analysis. Participants only completed one start in each condition. However, this is consistent with race conditions where swimmers only have one opportunity to complete their start. Furthermore, data from our previous research reveals inter-start variability is low when the backstroke ledge is used (1.98% coefficient of variation for time to 10 m) [3]. We did not account for buoyancy or drag when calculating predicted takeoff velocities. Although some participants’ hips were partially submerged prior to the start, the submerged volume was small so we expect that the impact of this approach was negligible. Similarly, we expect that the influence of drag on the predicted horizontal takeoff velocity was small.

Findings from this study suggest that selecting a setup position with the lower-limbs more flexed or more extended does not affect overall performance. However, this finding was based on the pooled data of all participants. Individually, some participants had differences in time to 10 m exceeding 0.1 s; some achieved a shorter time to 10 m in the flexed start position while others achieved a shorter time to 10 m in the extended start position (Appendix A, Table A1). Accordingly, it appears that the optimal setup positions may vary between swimmers, and that coaches and athletes should determine which setup position results in better performance on an individual basis. Individual characteristics may provide some indication as to which position may be more appropriate. For example, swimmers who can generate more muscular power may find the flexed start position more suitable, as they may take advantage of the greater horizontal takeoff velocity. In contrast, swimmers who have produce less power may find the extended setup position where they can take advantage of decreased drag during the flight phase is more appropriate. Building upon our previous research [3,17], the negative correlation between head entry distance and time to 10 m indicates that head entry distance continues to be a valuable, inexpensive tool for coaches to quickly estimate start performance when comparing different start techniques.

## Figures and Tables

**Table 1:** Means and respective standard deviations for kinematic data (variables calculated from digitized landmark positions during the start) in the flexed and extended test conditions.

Variable	Flexed	Extended	*p* Value	Effect Size (d_z_)
Average hip angular velocity (°/s)	620 ± 100	620 ± 96	0.37	−0.11
Average knee angular velocity (°/s)	310 ± 29	290 ± 43	0.08	0.49
Time of hip extension onset (s)	0.19 ± 0.06	0.20 ± 0.04	0.32	−0.15
Time of knee extension onset (s)	0.22 ± 0.07	0.24 ± 0.08	0.02 *	−0.75 ^‡^
Hip angle during setup (°)	62 ± 11.4	60 ± 15	0.26	−0.22
Knee angle during setup (°)	46 ± 11	64 ± 13	<0.01 *	4.8 ^†^
Hip height above water during setup (m)	0.12 ± 0.07	0.16 ± 0.08	<0.01 *	1.09 ^†^
Head entry distance (m)	2.1 ± 0.21	2.2 ± 0.25	0.07	−0.53 ^‡^
Time to 10 m (s)	5.02 ± 0.50	5.03 ± 0.49	0.36	−0.12

* indicates a statistically significant difference (*p* < 0.05). ^†^ indicates a large (d_z_ > 0.8) effect size and ^‡^ indicates a medium (0.8 > d_z_ > 0.5) effect size.

**Table 2:** Means and respective standard deviations for kinetic data (variables calculated from force data from the load cell and force plate) in the flexed and extended test conditions.

Variable	Flexed	Extended	*p* Value	Effect Size (d_z_)
Hand contact time (s)	0.37 ± 0.06	0.35 ± 0.04	0.16	0.33
Hands horizontal impulse (Ns)	−83 ± 52	−91 ± 78	0.28	0.19
Hands vertical impulse (Ns)	67 ± 29	53 ± 23	0.02 *	0.81 ^†^
Foot / total contact time (s)	0.66 ± 0.09	0.64 ± 0.07	0.14	0.37
Feet horizontal impulse (Ns)	330 ± 77	320 ± 90	0.34	0.13
Feet vertical impulse (Ns)	250 ± 64	250 ± 72	0.49	-0.01
Feet peak rate of force development (N/s)	38 ± 18	36 ± 20	0.32	0.15
Horizontal takeoff velocity (m/s)	3.5 ± 0.37	3.3 ± 0.34	0.03 *	0.66 ^‡^
Vertical takeoff velocity (m/s)	0.66 ± 0.21	0.58 ± 0.23	0.11	0.42
Net takeoff velocity (m/s)	3.6 ± 0.37	3.4 ± 0.36	0.02 *	0.78 ^‡^
COM takeoff angle (°)	11 ± 3.4	10 ± 4.3	0.26	0.21

* indicates a statistically significant difference (*p* < 0.05). ^†^ indicates a large (d_z_ > 0.8) effect size and ^‡^ indicates a medium (0.8 > d_z_ > 0.5) effect size.

**Table 3:** Means and respective standard deviations for EMG data (variables calculated from voltage data from sEMG sensors) in the flexed and extended test conditions.

Variable	Flexed	Extended	*p* Value	Effect Size (d_z_)
Glute activity during setup (%MVC)	5.6 ± 8.0	7.5 ± 9.8	0.15	−0.36
Vastus activity during setup (%MVC)	8.6 ± 6.9	21 ± 13	<0.01 *	−1.05 ^†^
Glute activity onset (s)	0.36 ± 0.06	0.35 ± 0.07	0.41	0.07
Vastus activity onset (s)	0.30 ± 0.05	0.28 ± 0.05	0.08	0.49
Peak glute activity during push (%MVC)	86 ± 40	79 ± 48	0.19	0.29
Peak vastus activity during push (%MVC)	120 ± 57	110 ± 50	0.08	0.48
Time to peak glute activity (s)	0.56 ± 0.06	0.54 ± 0.10	0.29	0.19
Time to peak vastus activity (s)	0.55 ± 0.06	0.51 ± 0.06	0.14	0.39
Glute rate of force development (%MVC/s)	560 ± 220	550 ± 240	0.40	0.08
Vastus rate of force development (%MVC/s)	580 ± 330	510 ± 250	0.12	0.39

* indicates a statistically significant difference (*p* < 0.05). ^†^ indicates a large (d_z_ > 0.8) effect size.

## Appendix A

**Table A1:** Kinematic data for individual participants *.

Participant and Condition	Average Hip Angular Velocity (°/s)	Average Knee Angular Velocity (°/s)	Time of Hip Extension Onset (s)	Time of Knee Extension Onset (s)	Hip Angle during Setup (°)	Knee Angle during Setup (°)	Hip Height above Water during Setup (m)	Head Entry Distance (m)	Time to 10 m (s)
1 Extended	620	310	0.17	0.16	72	46	0.12	2.0	5.18
1 Flexed	620	320	0.17	0.17	70	35	0.12	2.2	5.15
2 Extended	770	250	0.27	0.21	46	60	0.057	2.5	4.71
2 Flexed	820	280	0.25	0.19	53	40	0.039	2.4	4.68
3 Extended	670	240	0.19	0.23	52	60	0.12	2.1	5.42
3 Flexed	660	280	0.17	0.23	62	36	0.071	2.0	5.57
4 Extended	490	370	0.18	0.32	47	62	0.076	2.2	5.53
4 Flexed	500	320	0.17	0.21	44	41	0.074	2.1	5.55
5 Extended	530	280	0.21	0.17	89	58	0.26	2.2	5.37
5 Flexed	530	330	0.13	0.14	79	41	0.18	2.0	5.41
6 Extended	610	270	0.19	0.27	62	84	0.16	2.2	5.37
6 Flexed	580	290	0.21	0.25	65	64	0.14	2.1	5.25
7 Extended	670	270	0.25	0.21	79	57	0.25	2.2	4.20
7 Flexed	680	310	0.17	0.17	78	43	0.21	2.1	4.23
8 Extended	680	310	0.13	0.19	55	58	0.21	2.2	4.69
8 Flexed	610	370	0.16	0.20	54	37	0.10	2.0	4.62
9 Extended	700	260	0.21	0.23	55	76	0.26	2.5	4.38
9 Flexed	710	280	0.17	0.21	60	55	0.23	2.4	4.33
10 Extended	480	340	0.24	0.44	51	85	0.10	1.6	5.41
10 Flexed	480	300	0.35	0.40	54	66	0.068	1.7	5.38

* Participants 2, 7 and 9 were male. The remaining participants were female.

## Appendix B

**Table A2:** Kinetic data for individual participants *.

Participant and Condition	Hand Contact Time (s)	Hands Horizontal Impulse (Ns)	Hands Vertical Impulse (Ns)	Feet Horizontal Impulse (Ns)	Foot/Total Contact Time (s)	Feet Vertical Impulse (Ns)	Feet Peak Rate of Force Development (N/s)	Horizontal Takeoff Velocity (m/s)	Vertical Takeoff Velocity (m/s)	Net Takeoff Velocity (m/s)	COM Takeoff Angle (°)
1 Extended	0.30	−64	47	0.70	280	210	40	3.2	0.46	3.3	8.2
1 Flexed	0.35	−60	51	0.79	300	240	40	3.6	0.47	3.6	7.5
2 Extended	0.39	−270	90	0.77	540	400	50	3.0	1.04	3.2	19
2 Flexed	0.37	−170	80	0.66	470	370	60	3.3	0.81	3.4	14
3 Extended	0.39	−180	26	0.69	390	340	27	2.9	0.60	2.9	12
3 Flexed	0.37	−170	41	0.73	400	340	48	3.1	0.69	3.2	13
4 Extended	0.38	−58	66	0.62	250	200	11	3.4	0.84	3.5	14
4 Flexed	0.38	−46	91	0.66	230	180	21	3.3	1.01	3.5	17
5 Extended	0.31	−60	16	0.59	220	220	45	3.0	0.31	3.0	6.0
5 Flexed	0.28	−35	26	0.54	220	180	11	3.4	0.37	3.4	6.2
6 Extended	0.36	−98	52	0.59	300	240	11	3.2	0.48	3.3	8.5
6 Flexed	0.38	−52	44	0.70	274	220	15	3.5	0.49	3.5	7.9
7 Extended	0.35	−90	47	0.64	330	300	76	3.3	0.58	3.4	10
7 Flexed	0.33	−91	54	0.61	340	280	62	3.5	0.48	3.5	7.9
8 Extended	0.29	−3.2	44	0.52	270	200	36	3.7	0.47	3.8	7.2
8 Flexed	0.31	−62	81	0.55	320	200	34	3.6	0.79	3.7	12
9 Extended	0.36	−16	78	0.58	310	260	44	3.9	0.74	4.0	11
9 Flexed	0.42	−33	120	0.63	370	290	53	4.5	0.80	4.6	10
10 Extended	0.39	−75	66	0.70	310	170	20	3.8	0.30	3.8	4.5
10 Flexed	0.49	−110	84	0.78	320	230	40	3.5	0.78	3.6	13

* Participants 2, 7 and 9 were male. The remaining participants were female.

## Appendix C

**Table A3:** EMG data for individual participants *.

Participant and Condition	Glute Activity during Setup (%MVC)	Vastus Activity during Setup (%MVC)	Glute Activity Onset (s)	Vastus Activity Onset (s)	Peak Glute Activity during Push (%MVC)	Peak Vastus Activity during Push (%MVC)	Time to Peak Glute Activity (s)	Time to Peak Vastus Activity (s)	Glute Rate of Force Development (%MVC/s)	Vastus Rate of Force Development (%MVC/s)
1 Extended	0.76	7.5	0.25	0.36	40	49	0.53	0.49	500	300
1 Flexed	4.9	1.5	0.27	0.44	41	54	0.62	0.56	560	290
2 Extended	0.03	12	0.22	0.51	54	58	0.71	0.52	640	310
2 Flexed	0.22	2.3	0.23	0.39	44	93	0.60	0.43	310	490
3 Extended	3.0	4.7	0.24	0.42	54	62	0.63	0.43	540	840
3 Flexed	0.81	5.1	0.29	0.27	70	79	0.50	0.51	530	640
4 Extended	4.2	2.9	0.32	0.40	49	64	0.61	0.58	450	340
4 Flexed	4.2	3.4	0.32	0.38	63	63	0.58	0.58	670	440
5 Extended	21	30	0.30	0.28	170	120	0.51	0.57	680	334
5 Flexed	4.3	11	0.24	0.27	170	150	0.48	0.52	780	500
6 Extended	6.8	29	0.30	0.31	74	120	0.58	0.55	240	360
6 Flexed	6.0	16	0.34	0.37	60	100	0.60	0.54	230	430
7 Extended	3.1	25	0.31	0.38	140	190	0.52	0.52	1000	810
7 Flexed	1.2	6.3	0.29	0.39	110	160	0.51	0.46	910	890
8 Extended	30	42	0.23	0.29	110	170	0.39	0.37	790	950
8 Flexed	28	5.3	0.30	0.33	120	250	0.45	0.47	720	1400
9 Extended	4.8	34	0.32	0.35	69	130	0.51	0.55	400	430
9 Flexed	3.1	13	0.35	0.41	100	150	0.60	0.62	530	500
10 Extended	1.6	24	0.37	0.30	22	96	0.38	0.53	250	450
10 Flexed	3.4	23	0.40	0.42	80	120	0.62	0.77	370	280

* Participants 2, 7 and 9 were male. The remaining participants were female.
